# Gastroduodenal Changes Two Years After Eradication of Helicobacter pylori in a Population-Based Cohort

**DOI:** 10.14740/gr646w

**Published:** 2015-04-03

**Authors:** Stefan Redeen, Fredrik Petersson, Stergios Kechagias, Jens Rehfeld, Kurt Borch

**Affiliations:** a Department of Surgery and Department of Clinical and Experimental Medicine, Linkoping University, 58185 Linkoping, Sweden; b Department of Pathology, National University Health System, 119074 Singapore, Singapore; c Department of Clinical Biochemistry, Rigshospitalet, University of Copenhagen, Denmark

**Keywords:** Duodenitis, Follow-up, Gastrin, Gastritis, *Helicobacter pylori*, Metaplasia, Somatostatin, Symptoms

## Abstract

**Background:**

The main cause of chronic gastritis is *Helicobacter pylori (H. pylori)* infection. Some individuals with *H. pylori*-related chronic gastritis develop atrophy of the gastric mucosa, a risk factor for gastric neoplasia. When *H. pylori*-associated gastritis is encountered, it is important to be aware of its natural history and reversibility of associated histopathological and hormonal changes.

**Methods:**

A sample of 501 volunteers from the general population in the municipality of Linkoping, Sweden, was examined with esophago-gastro-duodenoscopy (EGD) with biopsy. Blood samples were collected in the fasting state and the subjects answered a questionnaire concerning lifestyle factors, medications and disease history. At a primary follow-up examination, after 8 years, 314 participants were re-examined and those infected with *H. pylori* received eradication. Two years after successful eradication therapy, 82 participants attended re-examination with EGD and blood sampling, as in the previous examinations.

**Results:**

In this prospective cohort study of a sample of volunteers from the general population, all of the 82 participants had chronic gastritis with at least one positive *H. pylori* test before eradication therapy. During follow-up, non-steroid-inflammatory-drug (NSAID) use had decreased significantly (P = 0.007, McNemar). The *H. pylori* serology was still positive in 79/82 subjects (P = 0.007, McNemar). The basal gastrin and cholecystokinin (CCK) concentrations both had decreased (P < 0.001 for both, Wilcoxon), whereas the P-somatostatin had increased (P < 0.001, Wilcoxon). Symptoms included in the self-administered symptom questionnaire concerned the last 3 months showed no big difference at all. The inflammation had decreased in both antrum (before 2/38/42/0 and after 60/22/0/0, P < 0.0001) and corpus (before 3/54/22/3 and after 58/23/1/0, P < 0.0001). Changes in the inflammatory activity had decreased significantly in both the antrum (P < 0.001) and the corpus (P < 0.001). Intestinal metaplasia was without changes. Regarding the duodenal bulb, the inflammation decreased.

**Conclusions:**

Being aware of the natural history of chronic gastritis, even beyond eradication of *H. pylori*, is important because of the associations to gastric neoplasia and ulcer disease. The blood mirror of gastroduodenal parameters showed decreased values, except for somatostatin that increased and a symptomatology with no significant changes although, morphologically determined, both inflammation and atrophy had decreased.

## Introduction

More than half of the world’s population are colonized with *Helicobacter pylori (H. pylori)* in the upper gastrointestinal tract, mainly the gastric mucosa. Most of these persons have no symptoms of the infection, and only a few (15%) will develop *H. pylori*-related chronic gastritis, although there is a subclinical cascade of changes due to the infection [1].

In 2005, Warren and Marshall were awarded the Nobel Prize for discovery of the spirochete *H. pylori* and its role in gastritis and ulcer disease [2, 3]. The common opinion today is that the infection usually is acquired in early childhood and that it, initially, may be associated with symptomatic acute gastritis and even ulcer in young people [4]. Transmission of *H. pylori* is thought to be mainly from person to person via faecal-oral or oral-oral routes. It may also be transmitted by contaminated water supply. In approximately 80% of *H. pylori*-infected subjects, chronic gastritis will occur. At present, the prevalence of *H. pylori* is decreasing in the Western World [5] but not in developing countries. People on regular treatment with NSAIDs, including, aspirin, are at risk of developing chronic gastritis [6].

### Natural history of chronic gastritis

It is well documented that the majority of *H. pylori*-infected will develop chronic gastritis [7, 8]. It is important to understand the subclinical natural history of chronic gastritis, including the post eradication changes. Of particular importance is to understand the development and dynamics of atrophic gastritis, because it is a major risk for gastric neoplasia [9-11]. Having a young patient with uncomplicated atrophic gastritis raises the question whether regular screening with gastroscopy is indicated [10]. Awareness of a probable increase of the incidence of pancreatic neoplasia in atrophic gastritis associated with pernicious anaemia is also important [12].

The prevalence of atrophic gastritis in the general population has been assessed by screening gastric function. Specifically, screening involves analysis of S-pepsinogen I (PGI) or the S-pepsinogen I/S-pepsinogen II ratio (PGI/PGII), with subnormal levels indicating atrophy of the gastric corpus mucosa [13]. Screening with these surrogate markers shows an overall prevalence of atrophy of the corpus mucosa among adults in Europe and New Zealand of 3-6% increasing with age [14-16]. Using histology, however the prevalence of atrophy of the corpus mucosa ranges 10-37% in the general population in northern Europe [1, 17-20].

Several studies have documented the natural history of chronic gastritis in patient series [21-24], but there are few population-based studies [23, 25, 26]. The progression of *H. pylori*-associated chronic non-atrophic gastritis to atrophic gastritis is slow, and long-term observation is needed to obtain reliable data [27-29]. However, the longer the observation intervals are, the fewer the number of subjects available for follow-up. This is of particular concern amongst the elderly, who have the highest incidence of mucosal atrophy.

### Post *H. pylori* eradication

There is little knowledge about post eradication functional and morphological changes. This study investigates a population, previous examined with gastroscopy including biopsy, histological examination and a standardized symptom questionnaire 2 years post eradication regarding gastric function, symptomatology, and morphology.

The aim of this study was to evaluate symptomatology, morphology and gastroduodenal function in a previously studied subpopulation from the general population, 2 years after *H. pylori* eradication.

## Material and Methods

### Ethics

The study was performed in accordance with the Helsinki Declaration and was approved by the Local Ethics Committee. Informed written consent was obtained from all participants. The approval was for baseline investigation and follow-up in the study. Ethical issues included the discomfort of performing gastroscopy of healthy volunteers and how to handle the findings at gastroscopy.

### Study population

At the initiation of the study, 2,000 persons (20 men and 20 women for each year of the ages 35 - 85 years) randomly selected from the population register of the municipality of Linkoping, Sweden, were invited; 501 accepted and underwent oesophago-gastro-duodenoscopy (EGD) with biopsy and filled in a questionnaire about lifestyle, medications and disease history.

At the first follow-up examination, 314 participants were examined, and at this finally examination, 2 years after successful *H. pylori* eradication therapy was given 82 participants were possible to investigate in. Regarding the present study, inclusion criteria were presence of chronic gastritis, and occurrence of at least one positive *H. pylori* test (serology, histology, urea breath test (UBT), urease test on fresh biopsy). Successful eradication therapy was verified with UBT (^13^CO_2_-UBT (ref. limit < 3.5 per mille)). Table 1 presents the population in this study.

**Table 1:** Background Variables

	Female	Male	Total
N	35	47	82
Follow-up time after eradication	-	-	29.8 months (23.6 - 145.2)
Follow-up time, person years			269.6
Median age at eradication treatment (range)	70.0 (41.0 - 81.8)	66.5 (51.3 - 86.8)	68.9 (41.0 - 86.8)
History of ulcer in total			17 (baseline 9, first follow-up 3, present study 5)

Eradication therapy consisted of esomeprazol 20 mg, two amoxicillin 500 mg and one clarithromycin 500 mg twice a day in a week. All participants were examined in a standardized way previously described with gastroscopy including biopsy for morphological examination, blood tests and a questionnaire about symptoms during last 3 months at the primary and the follow-up investigations. UBT was done in all participants 1 month after eradication therapy to confirm a successful eradication therapy. The questionnaire included question about lifestyle and medication, i.e. non-steroid-inflammatory-drug (NSAID). Five of the 82 participants experienced diarrhea during treatment. None of these required treatment.

### Gastroscopy

The volunteers fasted for at least 6 h before the examination. Blood samples were drawn and EGD was carried out after pharyngeal anesthesia with lidocaine spray (Xylocaine, Astra, Sodertalje, Sweden). High resolution endoscopy was not used. The Olympus GIF-100 gastroscope was used and biopsies were taken with the same forceps design in all subjects at baseline and at follow-up examinations. Sedation with 2 - 3 mg intravenous flunitrazepam intravenously (dormicum, Roche AB, Stockholm, Sweden) was given on demand. Three biopsy specimens for histological examination were routinely collected from gastric body (major, anterior, and posterior aspect) and the antrum (within 3 cm of the pylorus). One additional biopsy specimen from each location was analyzed for *H. pylori* by urease test (CLO-test, Delta West Pty Ltd, Bentley, Australia), which was considered to indicate the presence of *H. pylori* if positive within (3 h paper I) 20 min, 1 h, 3 h and 12 h at follow-up. A further biopsy specimen from each of the corpus and antrum was collected for culture of *H. pylori*.

### Histological examination and *H. pylori* status

An experienced pathologist blinded to other data from the study performed the microscopic examination at baseline and at follow-up after *H. pylori* eradication therapy in the same standardized manner.

After orientation, fixation in neutral formaldehyde, and routine processing of the biopsies (three biopsies from the corpus, antrum and second part of the duodenum), sections cut (5 μm thick) perpendicular to the surface the biopsies were stained with hematoxylin-eosin (inflammation), Alcian blue-periodic acid-Schiff (mucus), and Giemsa stain (*H. pylori*).

The density of *H. pylori*, chronic inflammatory (lympho-plasmocytic) infiltrate, inflammatory activity (polymorphonuclear cells), glandular atrophy and intestinal metaplasia were scored (0: none, 1: mild, 2: moderate, or 3: severe) according to the Sydney system. When inflammation or atrophy was present in both the antrum and corpus, gastritis was classified as antrum- or corpus-predominant if there was a two-grade or greater difference between the scores for inflammation (or atrophy), and as pan-gastritis when there was less than a two-grade difference. Gastritis limited strictly to the antrum or corpus was classified accordingly as antrum- or corpus-predominant. The severity scores for gastritis are equal to the highest score for inflammation (or atrophy) in the antral or corpus mucosa.

Results from the first examination in this study also included the glandular atrophy of the gastric corpus mucosa, moderate to severe atrophy. Atrophy of the duodenal mucosa was histologically classified according to Alexander, and in the present study it was defined as grade III or grade IV.

### Blood analyses

At baseline and at first follow-up fasting blood samples were collected immediately preceding EGD. The samples were stored at -80 °C pending analysis. Pepsinogens in serum were measured with a sandwich enzyme immunoassay (ELISA) utilizing a pepsinogen I (PGI) and pepsinogen II (PGII) specific capture antibody, respectively, and a horseradish peroxidase (HRP) detection antibody (from GastroPanel^®^, Biohit Diagnostics, Helsinki, Finland). There is no cross-reactivity between the two assays. The reference interval is 30.0 - 120.0 µg/L for PGI, 3.0 - 10.0 µg/L for PGII and 3.0 - 20.0 for the ratio PGI/PGII. Values of PGI/PGII lower than 3.0 were considered as indicative of significant atrophy of the gastric corpus mucosa.

Serum IgG antibodies to *H. pylori* were analyzed by ELISA as described previously and reported as relative OD, that is, as a percent of positive standards (normal upper limit, 5%).

For measurement of α-amidated gastrins we used antiserum no. 2604 which is directed against the C-terminal sequence of the bioactive gastrins. The antiserum binds gastrin-71, -34 and -17 with equimolar potency, and negligible reactivity with cholecystokinins (CCKs) [30].

The CCK specific antiserum no. 92128 was used for the measurement of α-amidated and O-sulfated CCK peptides. The assay measures CCK-58, -33, -22 and -8 with equimolar potency [31]. For measurement of somatostatin we used antiserum no. R37 which binds somatostatin-14 and -28 with equimolar affinity [32].

The detection limit for all the immunoassay measurements is 0.5 pmol/L or less. P-gastrin, P-CCK and P-somatostatin are presented in picomol/L.

### UBT

^13^CO_2_-UBT was performed as in clinical routine in a VG ISOCHROM-µG mass spectrometer (Fisons, UK). Breath samples were taken before and 15, 30, 45 and 60 min after ingestion of 50 mg ^13^C urea. The result used is from the cumulative curve at 30 min (delta over baseline 30) with an upper limit of 3.5 per mille. The participants were instructed to avoid proton pump inhibitors (PPIs) 2 weeks before the examination. Those in need of PPI, e.g. for gastro-oesophageal reflux disease, were prescribed low dose H2-blockers during the 2 weeks preceding UBT. UBT was used 2 years after eradication. UBT was done after eradication therapy was given, and the results were successful in all included patients in this study.

### Statistical analyses

The MacNemar test for the significance of changes was used for categorical data and differences regarding continuous data were tested with the Mann-Whitney U test. A P-value of less than 0.05 was considered as significant.

## Results

In the present study from the general population, all of the 82 participants had chronic gastritis with at least one positive *H. pylori* test from the primary examination. At the follow-up examination, successful *H. pylori* eradication therapy was given. The UBT results were negative for all participants 2 years after eradication therapy. At the time of treatment, the median age was 68.9 (41.0 - 86.8) years and the median follow-up time after treatment was 29.8 (23.6 - 145.2) months (269.6 person years). Seventeen persons had a history of ulcer disease.

Demographic data are given in Table 1. All hormonal values from baseline and at follow-up are given in Table 2. The NSAID consumption decreased significant (P = 0.007, McNemar). The *H. pylori* serology was still positive in 79/82 subjects (P = 0.007, McNemar). The PGI/PGII ratio decreased significantly (P = 0.001, McNemar). The P-total gastrin decreased significantly (P < 0.001 for both, Wilcoxon), whereas the P-somatostatin and P-CCK had increased significantly (P < 0.001 for both, Wilcoxon).

**Table 2:** Hormonal Changes Observed at Baseline and at Follow-Up After *H. pylori* Eradication

N = 82	Baseline	Follow-up	P
NSAID use: at least weekly, including low dose	19/59	5/10	0.007
Post Hp-serology	0	68	0.001
PGI/PGII < 3.0	0	3	0.001
P-gastrin > 50	1	6	0.625
Urea breath test > 2.5	0	1	< 0.0001
P-CCK, median (range)	0.7 (0.1 - 2.5)	0.1 (0.1 - 1.9)	< 0.0001
P-gastrin, median (range)	9.0 (1.0 - 214)	6.0 (3.0 - 1,242)	< 0.0001
P-somatostatin, median (range)	6.0 (0.0 - 32.0)	13.0 (0.0 - 36.0)	< 0.0001

Chronic gastritis at histology and at least one positive *H. pylori* test before eradication of *H. pylori*, McNemar.

All symptoms are presented in Table 3 from the standardized and previously used questionnaire. Symptoms included in the self-administered symptom questionnaire concerned the last 3 months, preceeding EGD at baseline before eradication therapy and at follow-up 2 years after eradication therapy. The frequency of symptoms was graded into: none (0), not every month (1), every month (2), every week (3), and every day (4). The frequency of epigastric pain had decreased significantly (P = 0.028, McNemar), but there was no significant change in the frequency of other symptoms such as food intolerance, vomiting, acid regurgitation, heart burn, dysphagia, nausea, diarrhea, constipation, bloating and disturbing abdominal gas. None had severe problems or died. The only result regarding symptoms with some significance was epigastric pain, although in few participants. Five participants had relatively mild diarrhea after eradication therapy was given, one needed treatment, and everybody was healthy again.

**Table 3:** Abdominal Symptoms at Baseline and Follow-Up

	Baseline, yes/no	Follow-up, yes/no	P, Wilcoxon
Abdominal pain total	24/68	16/68	0.116 NS
Epigastric pain	7/68	2/68	0.028
Lower abdominal pain	15/64	12/64	0.503 NS
Acid regurgitation	45/72	43/72	0.549 NS
Acid reflux	40/72	39/72	0.839 NS
Dysphagia	8/72	10/72	0.782 NS
Nausea	24/72	28/72	0.698 NS
Vomiting	11/72	11/92	> 0.999 NS
Bloating after meal	35/71	38/71	0.322 NS
Bloating general	27/71	38/71	0.723 NS
Gas	53/72	60/72	0.111 NS
Constipation	40/70	41/70	0.838 NS
Diarrhea	41/71	40/71	0.860 NS

In the study population in frequency of never, not every month, every month, but not every week, every week, but not every day, every day and here in total. Symptoms that last 3 months.

The morphologies both in antrum and corpus are presented in Table 4. The inflammation had decreased in both antrum (before 2/38/42/0 and after 60/22/0/0, P < 0.0001) and corpus (before 3/54/22/3 and after 58/23/1/0, P < 0.0001) after eradication of *H. pylori*. Changes in the inflammatory activity had decreased significantly in both the antrum (P < 0.001) and the corpus (P < 0.001). Regarding intestinal metaplasia there were no significant changes. Chronic inflammation had decreased significantly in both the antrum (before 2/38/42/0 and after 60/22/0/0, P < 0.0001) and the corpus (3/54/22/3 and after 58/23/1/0, P < 0.0001) 2 years after eradication treatment. Atrophy had decreased significantly in the antrum, but was unchanged in the corpus. Inflammatory activity had decreased significantly in both the antrum (P < 0.001) and corpus (P < 0.001).

**Table 4:** Morphology

N = 82	Antrum 2, 0-3/N	Antrum 3, 0-3/N	P	Corpus 2, 0-3/N	Corpus 3, 0-3/N	P
Chronic inflammation	0/2 1/38 2/42 3/0	0/60 1/22 2/0 3/0	< 0.0001	0/3 1/54 2/22 3/3	0/58 1/23 2/1 3/0	< 0.0001
Atrophy	0/60 1/18 2/1 3/2	0/72 1/8 2/0 3/1	0.003	0/64 1/10 2/7 3/1	0/74 1/5 2/1 3/2	0.014
Inflammatory activity	0/12 1/56 2/14 3/0	0/79 1/3 2/0 3/0	< 0.0001	0/24 1/47 2/10 3/1	0/80 1/1 2/1 3/0	< 0.0001
Intestinal metaplasia	0/58 1/21 2/1 3/2	0/64 1/15 2/2 3/1	0.142	0/74 1/5 2/3 3/0	0/72 1/9 2/0 3/1	0.734

Antrum and corpus at first (2) and second (3) follow-up. These morphological features are semi-quantitatively assessed on a four-graded scale (visual analogue scale) equivalent to verbal description none = 0, mild = 1, moderate = 2 and severe = 3. In total 82 persons were investigated; it was not enough material for analysis at all locations.

Table 5 shows the results presented from the duodenal bulb: 66 of 77 subjects had score 0 for inflammation before eradication therapy. At the 2-year follow-up examination, the corresponding frequency was 74 of 77 (P = 0.013, McNemar). Bulbitis was decreased after eradication. Regarding gastric metaplasia, there were no significant changes after eradication therapy.

**Table 5:** Duodenal Inflammation and Gastric Metaplasia at First (2) and Second (3) Follow-Up

N = 77	B 2, 0-3	B 3, 0-3	P, McNemar
Bulbitis	0/66, 1/6, 2/4, 3/1	0/74, 1/3, 2/0, 3/0	0.013
Gastric metaplasia	0/53, 1/25, 2/4, 3/0	0/46, 1/25, 2/6, 3/0	0.171

The visual analogue scale 0 - 4 was used.

## Discussion

Chronic gastritis is common worldwide. If there are benefits with eradicating the *H. pylori* infection and related risk conditions regarding morbidity and mortality, the general population should be offered this.

In this study, we focused on gastric function, symptomatology and morphology after successful eradication therapy. It is well known that the *H. pylori* prevalence has decreased in the Western world. The distribution in developing countries is mainly unknown.

The follow-up period, for other studies, could include more patients and even from different countries during a longer study period. We had almost 270 person years in total as a basis for follow-up our study population.

We found changes regarding NSAID consumption which had decreased at follow-up, and the reason to decreased NSAID consumption is beyond this study. Maybe awareness of complications related to NSAID use now is common and other drugs are used instead. Plasma CCK and gastrin concentrations decreased in the follow-up period, whereas somatostatin concentrations increased. These changes can all be related to decreased inflammation of the gastric mucosa. Considering symptoms, there were no major differences.

Villako et al found interesting dynamic effects in an Urban Estonian society with gastritis affecting the body increased significantly with age and gastritis in antrum decreased [14]. The reason behind this is not obvious from this study, but shows that development exists and is important to understand.

Further from the Maastricht III consensus report with management of *H. pylori* infection one comment is that the burden of gastric cancer is considerable but varies geographically [6]. Weather eradication therapy should be given in asymptomatic *H. pylori* infection is still a matter of debate.

Follow-up studies like this with symptoms, hormonal and morphological aspects are uncommon. Houghton et al conclude from the background that gastric cancer remains a leading cause of death worldwide that in the name of preventable disease it is important with effective eradication. Still the general population are not the target for eradication.

Of course it is important to decrease inflammation and activity of inflammation, and the clinical manifestations over time needs to be investigated in the future.

From other studies we know that somatostatin increases after eradication therapy indicating possible recovery of the mucosa [33].

The bulbitis was decreased after eradication therapy of *H. pylori*.

In conclusion, this study shows that gastric inflammation and activity in the inflammation decrease after eradication of *H. pylori*. Hormonal changes occur but there are no specific major changes in symptomatology.
